# Idiopathic pseudoaneurysmal omental bleeding, a rare cause of life-threatening acute abdomen

**DOI:** 10.1093/jscr/rjae048

**Published:** 2024-02-21

**Authors:** Zirong Yu, Mohammed Ali Alhamadani, Marthe Chehade, Danette B Wright

**Affiliations:** Department of Surgery, Princess Alexandra Hospital, Woolloongabba, Queensland, Australia; Department of Urology, John Hunter Hospital, New Lambton Heights, New South Wales, Australia; Department of Surgery, Westmead Hospital, Westmead, New South Wales, Australia; Clinical Network Director Surgery, Western Sydney Local Health District, Blacktown, New South Wales, Australia

**Keywords:** omental bleeding, acute abdomen, haemoperitoneum, pseudoaneurysmal rupture

## Abstract

Mesenteric aneurysms and their complications can be a life-threatening presentation of acute abdomen to the emergency department. The majority of mesenteric artery aneurysms are incidentally detected on imaging investigations and are asymptomatic. Symptomatic mesenteric aneurysms manifest as hemoperitoneum or abdominal pain. In addition, treatment of symptomatic aneurysms is delayed due to the infrequent consideration of the diagnosis in patients presenting with abdominal pain. Timely and accurate diagnosis is of paramount importance as any delay in definitive surgical management can lead to increased patient’s mortality and morbidity with up to 25% of mesenteric aneurysms may be complicated by rupture.

## Introduction

Mesenteric aneurysms and their complications can be a life-threatening presentation of acute abdomen to the emergency department [1]. Autopsy studies data suggest that mesenteric artery aneurysms may be more common than abdominal aortic aneurysms with a prevalence of mesenteric aneurysms estimated to be up to 10% and that of abdominal aortic aneurysms up to 0.5%. The prevalence of mesenteric artery aneurysms varies. Its distribution is as follows: ~70% of cases of aneurysms are in the splenic artery; 20% are in the hepatic artery; 5% are in the superior mesenteric artery; 6% are in the gastroduodenal artery and pancreatic branches; 4% are in the coeliac artery; 4% are in the gastric and gastroepiploic arteries; 3% are in the jejunal and ileocolic arteries; and 1% are in the inferior mesenteric artery. About one-third of the cases had multiple aneurysms. The majority of mesenteric artery aneurysms are incidentally detected on imaging investigations and are asymptomatic. Symptomatic mesenteric aneurysms manifest as hemoperitoneum or abdominal pain. In addition, treatment of symptomatic aneurysms is delayed due to the infrequent consideration of the diagnosis in patients presenting with abdominal pain.

Timely and accurate diagnosis is of paramount importance as any delay in definitive surgical management can lead to increased patient mortality and morbidity with up to 25% of mesenteric aneurysms may be complicated by rupture [2]. Haemoperitoneum due to omental aneurysmal haemorrhage as a cause for the acute abdomen can be due to trauma, neoplasia, vasculitis, coagulopathy, omental artery aneurysm/pseudoaneurysm, and idiopathic [3]. Haemoperitoneum secondary to pseudoaneurysmal omental bleeding is a very rare and life-threatening cause of acute abdomen necessitating urgent surgical management. Herein, we present a unique case of haemoperitoneum due to ruptured omental pseudoaneurysm in a patient on systemic anticoagulation and successful treatment with surgery.

## Case presentation

A 77-year-old man presented to the emergency department with acute left upper quadrant (LUQ) abdominal pain associated with nausea and dyspnoea that worsened despite analgesia. He reported no history of fevers, anorexia, vomiting, alteration in bowel habit, or urinary symptoms. His past medical history was significant with autoimmune limbic encephalitis, ischemic heart disease, congestive heart failure, hypertension, gout, asthma, obstructive sleep apnoea, and atrial fibrillation for which he takes Digoxin and Apixaban. He is an ex-smoker of 40 pack years and occasionally drinks alcohol.

**Figure 1:**
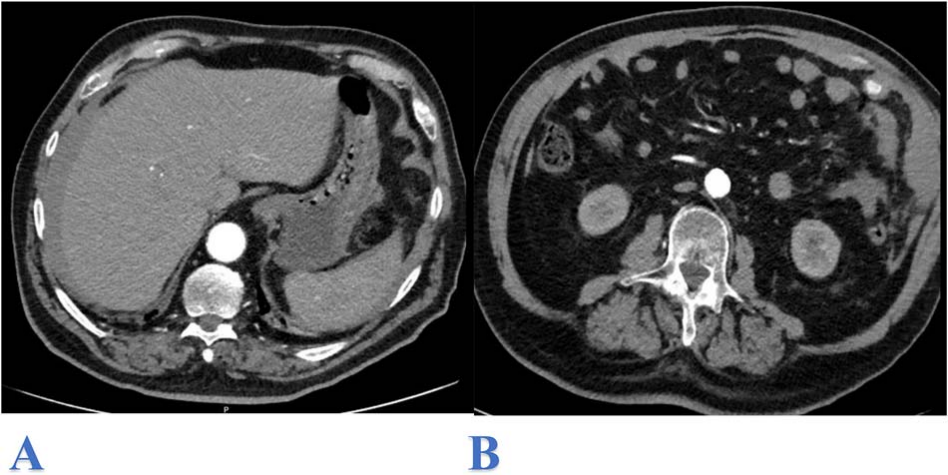
Axial CT abdomen image (arterial phase) showing: bilateral subphrenic hemoperitoneum (A) and contrast extravasation from the upper left lateral omentum (B).

On arrival to emergency, the patient was haemodynamically unstable with a blood pressure of 75/25 mmHg, heart rate of 71 bpm, respiratory rate of 30/min, and was afebrile. His Glasgow Coma Scale fluctuated between 14 and 15. Initial examination showed a diffusely distended abdomen that was tense to palpation with peritonism localized to the LUQ. Laboratory investigations showed haemoglobin of 146 g/L, white cell and platelet counts of 10.4 × 10^3^ and 171 × 10^3^, respectively. An urgent computed tomography (CT) scan of the abdomen showed moderate to large volume haemoperitoneum in the right and left subphrenic spaces and along the anterior and left lateral abdominal walls, extending into the pelvis. There was active contrast extravasation to the left anterolateral abdomen at the position of the greater omentum (Fig. 1A, B). Whilst in emergency, the patient deteriorated further, requiring vasopressors for haemodynamic support with a repeat of full blood count showing haemoglobin of 114 g/L. A massive transfusion protocol was initiated and his anticoagulated state was reversed with an off-label use of Prothrombinex as per haematology team advice.

**Figure 2:**
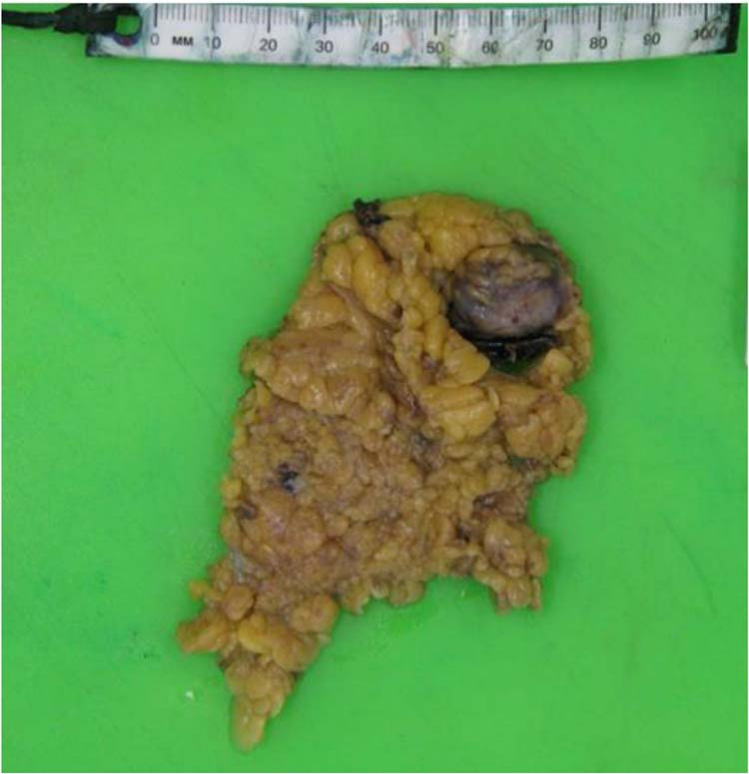
Macroscopic resected specimen of Omentum with bleeding pedicle.

The patient underwent an emergency exploratory laparotomy that exposed significant intraperitoneal haematoma. Intraoperative examination identified a haemorrhage from a portion of the omentum in the LUQ (Fig. 2) which was resected. A total 3 L of blood were evacuated from the peritoneal cavity. Postoperatively, the patient was admitted to the intensive care unit for 2 days and made gradual recovery. His hospitalization was complicated by hospital acquired pneumonia which was treated with intravenous antibiotics and was discharged after 2 weeks. Microscopic examination of the resected omental specimen showed features consistent with a pseudoaneurysm.

## Discussion

Omental bleeding is a rare cause of potentially life-threatening haemoperitoneum and haemorrhagic shock with mortality rate exceeding 30% [4]. Early diagnosis and definitive management are of a paramount importance in achieving favourable patient’s outcome. There are many causes of omental bleeding which includes trauma, neoplasia, anticoagulant medications, spontaneous arterial aneurysmal or pseudoaneurysmal rupture, omental torsion, vasculitis, and segmental arterial mediolysis [5]. Omental artery pseudoaneurysm is scarcely documented in the literature with a higher prevalence seen in men compared to women with a ratio of 6:1 and age of onset ranging widely from 20s to 80s [6].

Omental bleeding often presents as abdominal pain, especially over the epigastric area, and is accompanied with nausea, vomiting, diarrhoea, but can present with haemorrhagic shock and in severe cases, with abdominal compartment syndrome. Omental pseudoaneurysm can mimic other presentations of the acute abdomen with some case series mistakenly assessing omental bleeding as possible appendicitis or viscus perforation pre-operatively [4, 7].

Given the uncommon nature of this disease there are no specific recommendations for treatment. A recent literature review [5] found only 25 cases of omental bleeding in the literature, with varied management from transcatheter arterial embolization (TAE), laparotomy, or laparoscopy with omentectomy or simple ligation of the bleeding artery.

Diagnostic imaging is of critical importance in the evaluation of haemoperitoneum with omental artery pseudoaneurysm rupture largely depending on accurate imaging techniques such as ultrasonography (US) for the initial screening of hemoperitoneum and CT scans for more definitive diagnosis [8]. US can be exceptionally useful in facilitating rapid evaluation of hemoperitoneum in hemodynamically unstable patient [9]. CT scans with angiography however, is the imaging modality of choice in not only identifying the presence of hemoperitoneum and other intra-abdominal pathology but also in the localisation of the site of active vascular haemorrhage seen by contrast extravasation [10]. This is of critical importance in aiding the decision making for the surgeon or interventional radiologist in choosing the most appropriate management options for the patient [11].

There are varied strategies documented in the literature for the management of omental pseudoaneurysms. Conservative management with regular biochemical monitoring and anticoagulation reversal is limited to stable and clinically well patient [12]. Given reported cases of re-bleeding after trialling conservative management, aggressive therapy for definitive treatment is preferred [5, 6]. Specific management such as TAE have been reported to be effective, however for significantly unstable patients, laparotomy, or laparoscopy with omentectomy remains the mainstay for urgent surgical intervention [8].

Within the last several years, there have been multiple case reports within the literatures describing TAE as a treatment option for omental pseudoaneurysms [13]. Although TAE is a safe and minimally invasive procedure that can provide effective haemostasis when rapid haemostasis is required, it cannot provide omental tissue resection and hence it is difficult to make a pathological evaluation. One study reports a case of partial omentectomy 10 days post TAE of the left gastroepiploic artery due to suspicion of tumour in the bleeding omentum [11]. Furthermore, lack of TAE availability and experience with the technique probably is another barrier which limit its generalized application for management of mesenteric aneurysms and pseudoaneurysms.

Despite TAE being less invasive, the majority of patients treated for an omental pseudoaneurysm in the literatures underwent surgery with most patients receiving omentectomy and the remainder had either ligation or hematoma removal. No specific recommendations exist for follow up or surveillance for resected greater omental pseudoaneurysms. For most visceral aneurysms, recommendations include one-time cross-sectional imaging to identify and monitor the presence of concomitant aneurysms [13].
